# The male disadvantage in life expectancy: can we close the gender gap?

**DOI:** 10.1093/inthealth/ihaa106

**Published:** 2021-02-03

**Authors:** Muhammad Zakir Hossin

**Affiliations:** Department of Global Public Health, Karolinska Institute, SE-171 77, Stockholm, Sweden; Department of General Education, Eastern University, Dhaka, Bangladesh

**Keywords:** COVID-19, gender, lifestyle, longevity, men

## Abstract

Men are usually considered to be the stronger sex. However, when it comes to health, they are evidently weaker than their female counterparts. In almost all countries around the world, men consistently live shorter lives than women. The gender gap in longevity has once again been evident during the ongoing coronavirus disease 2019 (COVID-19) pandemic, which kills men disproportionately. Drawing on the relevant scientific literature and updated information, this article aimed to provide useful insights into the biological and social mechanisms that potentially underlie the gender gap in life expectancy.

## Introduction

Men are on average taller, more muscular and seemingly stronger than women and hence they are typically considered to be the stronger sex. Medical science, however, has a different story to tell us: men are biologically weaker than women. In almost all countries across the world, women outlive men^1^ and male mortality is higher for almost all primary causes of death.^2^ The female superiority in longevity is true not only for humans, but also for many other species of mammals.^3–5^

The natural sex ratio at birth is male biased, with 105 boys born on average for 100 girls on a global level. As the offspring grow up, males die in greater numbers than females at any given age,^2^ leading to a more balanced sex ratio in adult ages. The population sex ratio again reverses in old age, with women outnumbering men in most countries. Consequently, around 90% of all known supercentenarians (i.e. ≥110 years old) living on the planet are women.^4^ The ongoing coronavirus disease 2019 (COVID-19) pandemic further reminds us of the gender gap in mortality. In countries with available data, COVID-19 has been found to be killing more men than women.^6^

## The gender-health paradox

Although women live longer than men, paradoxically they tend to report poorer health and more frequent hospital visits. The diseases experienced by men and women differ in terms of their prevalence and severity.^2^ There is, however, no conclusive evidence in support of the hypothesis that women are sicker than men. It has been observed that women consistently suffer from increased morbidity in the domain of psychological health measures, but no increased risk is found with physical health conditions.^7^ Contrarily, men suffer more from severe and life-threatening illnesses like heart disease, stroke and cancer.^2,8^ These diseases are the major killers of our time, as well as the main culprits for premature deaths and the gender gap in mortality worldwide. A comprehensive study based on data from 45 developed countries^9^ demonstrated that women not only live longer than men, but also spend more years in good health. However, women spend more years in poor health relative to their overall life expectancy, implying that women's apparent morbidity disadvantage is directly linked to their longevity advantage.

## What causes the excess deaths in males?

Women and men are obviously different, both biologically and socially. The terms ‘sex’ and ‘gender’ are meant to describe those differences. While sex is a function of biology, gender is rooted in society. Women usually experience structural disadvantages in various spheres of the society, which limits their potential to maximize health and well-being. Yet they paradoxically seem to be the healthier sex. The mechanisms that underlie the gender gap in health and mortality are complex and not fully understood. Several biological and social mechanisms can be suggested as explanations.

### Biological mechanisms

From a biologic point of view, men are naturally programmed to die earlier than women at the very moment of conception. Available evidence indicates that the male foetus is biologically weaker and more vulnerable to maternal stress and pregnancy complications than the female foetus.^10,11^ This is clearly evident in the proportion of preterm births and the neonatal and infant mortality rates, which are higher in boys compared with girls.^12^ The sex differences at birth provide the foundation for the biologic explanation of male disadvantage in life expectancy.

Genetic disorders are often held responsible for excess morality in men. According to the heterogametic sex (or unguarded X) hypothesis, a damaged gene on the X chromosome can be naturally compensated by a similar gene in the second X chromosome in women, but not in men due to the lack of double X chromosomes.^13^ The higher number of infections, congenital disorders and deaths in male babies are possibly an expression of their lack of double X chromosomes.^8^ Furthermore, the mother's curse hypothesis states that the mitochondrial genome, which is believed to be exclusively inherited from the mother, may lead to male-specific harmful mutations in the mitochondria. The mitochondrial dysfunction is associated with ageing and a range of degenerative conditions in men.^3,14^

The sex hormones are argued to play a crucial role in the female advantage in longevity. The female sex hormone oestrogen is protective of cardiovascular diseases and is partly responsible for the lower incidence of such diseases in women until menopause. In contrast, the androgen hormone, which is higher in men, is associated with a higher risk of cardiovascular diseases.^3,8^ The stronger immune system of females is another factor that could contribute to the longevity gap.^2,8^ Female bodies are known to produce stronger immune responses and larger amounts of antibodies compared with male bodies.^15^ This offers females an enhanced capacity to fight off respiratory, bacterial and viral infections, including the deadly COVID-19.

Moreover, female bodies carry higher amounts of the good cholesterol (high-density lipoprotein), which protects against heart diseases.^3^ Men are disadvantaged even in the distribution of fat, because they usually accumulate excess fat around the stomach while women tend to carry excess fat in the hips and thighs.^8^ Any excess fat is harmful, but abdominal fat is more dangerous for cardiovascular health. The rate of telomere attrition, i.e. the progressive shortening of chromosomal length, is also known to be higher among adult men. The faster speed of telomere shortening in men is hypothesized to be associated with their accelerated aging and shorter lifespan,^3,8^ although the current evidence in support of this hypothesis is weak.^16,17^

The sexual selection hypothesis in evolutionary biology postulates that the male disadvantage in longevity is a trade-off between reproductive energy expenditure and physiological fitness.^18,19^ Interestingly, comparative animal studies suggest that the female superiority in longevity is more common in polygynous than monogamous species.^20^ The intense competition for mating opportunities and increased reproductive investment of the male sex may lead to their cumulative physiological deterioration and accelerate the longevity gap in favour of females.^18,19^ This hypothesis is evidentially supported by the observed enhanced survival of castrated males in mammalian species, including humans.^3,13^

### Social mechanisms

If biology were the sole cause behind the gender gap in life expectancy, one could expect the gap to be relatively constant over time and across societies. However, the gender gap in life expectancy varies considerably by time and context, suggesting that non-biological forces are operating to drive the trends. For instance, a boy born in Sweden today is expected to live 3.3 y less than a Swedish girl (81.7 vs 85.0 y) while the corresponding male–female gap in Russia is 10.5 y (67.6 vs 78.1 y).^1^ Thanks to medical advances and improved standards of living, global life expectancy dramatically increased in a linear fashion during the period from 1841 to 2000, with the best-performing life expectancy increasing by 3 months/y.^21^ However, it increased at a much slower rate in men than in women, resulting in a wider gender gap.

Men are more exposed to work-related stress and unhealthy behaviours, e.g. smoking and alcohol abuse, which are held responsible for their lower longevity.^22^ Further, men are typically disadvantaged by occupational hazards and so-called masculine behaviours that are highly risky and health hazardous. As a result, they die disproportionately in work-related accidents, car crashes, war and sporting activities.^23^ The male sex hormone testosterone has been shown to be responsible for predisposing men to health risk behaviours.^24^ This may be why research has found a link between marriage and increased life expectancy in men but not in women.^25^ Marriage may protect men from risky social habits, whereas women are less prone to risky behaviours regardless of their marital status.

Compared with the past, the gender gap in life expectancy has reportedly narrowed in most low-mortality countries in the last few decades.^26^ This is not surprising given that women have been increasingly entering the workforce and adopting health-damaging lifestyles like smoking and drinking. An interesting case in point is Sweden, where smoking is more prevalent in women than men^27^ and the gender gap in longevity is one of the lowest in Europe,^1^ although changes in other social and lifestyle factors and improved medical management of fatal diseases might also have propelled the trend of convergence in recent times.^28,29^

## Closing the gender gap

It is evident that women are the healthier sex and real champions in the final game of life. They have an innate biological advantage over men, with the female superiority in life expectancy appearing to be one of the most striking features of human biology. Biology is, of course, only a part of the story, since it cannot answer why the gender gap in life expectancy would fluctuate over time. Biology and society may interact with each other to produce gender-specific variations in diseases and mortality patterns. However, the relative influence of biology and society on the female advantage in life expectancy is an issue of debate.

The biological gap in life expectancy between women and men is a natural destiny that no fair society can avoid. On the contrary, the social gap in life expectancy is systematic and unjust. We can rarely change our genetic and biological make-ups and therefore cannot in principle eliminate the gender gap in longevity entirely. Even if biology accounts for a small part of the male's disadvantaged life span, gender equality in health may remain elusive. However, we can certainly narrow the gap by promoting healthy lifestyles and designing a society where both men and women will have a fair chance to maximize their health potentials.
